# Superior Vena Cava (SVC) Endovascular Reconstruction with Implanted Central Venous Catheter Repositioning for Treatment of Malignant SVC Obstruction

**DOI:** 10.3389/fsurg.2018.00004

**Published:** 2018-01-26

**Authors:** Stephanie Volpi, Francesco Doenz, Salah D. Qanadli

**Affiliations:** ^1^Cardio-Thoracic and Vascular Unit, Department of Radiology, Centre Hospitalier Universitaire Vaudois (CHUV), Lausanne, Switzerland

**Keywords:** superior vena cava syndrome, endovascular stenting, lung cancer, central venous catheter protection, vena cava obstruction

## Abstract

Superior vena cava (SVC) syndrome is a group of clinical signs caused by the obstruction or compression of SVC and characterized by edema of the head, neck, and upper extremities, shortness of breath, and headaches. The syndrome may be caused by benign causes but most of the cases are caused by lung or mediastinal malignant tumors. Stenting of SVC has become widely accepted as the palliative treatment for this condition in malignant diseases, as it offers rapid relief of symptoms and improves the quality of life. Preserving previously placed central venous catheters (CVCs) is a major issue in this population. We report the case of a patient with SVC syndrome caused by tumoral obstruction due to central small-cell lung cancer who had right subclavian implanted CVC and a preferential head and neck venous drainage through the left internal jugular and brachiocephalic vein (BCV). We describe a complex procedure of SVC reconstruction with two different objectives: left recanalization and stent placement to ensure head and neck venous drainage and right BCV stenting for CVC repositioning and subsequent replacement. We also review published cases of SVC obstructions stenting with catheter repositioning. The patient experienced quick relief of symptoms after treatment. Chemotherapy was rapidly delivered through the preserved implanted CVC access. A 3-month follow-up computed tomography showed stents patency.

## Introduction

Endovascular stent-based revascularization is used as a therapeutic measure in patients with superior vena cava (SVC) obstructions (1). It does not interfere with subsequent antitumor treatments and provides urgent relief of symptoms (2, 3). Additionally, stenting greatly improves the quality of life of patients (4–7) with a good long-term patency, especially in patients with benign causes (1). Revascularization in patients with malignant causes of obstructions aims of course to ensure venous drainage but also in several cases where the patient had or need the central venous catheters (CVCs) to preserve the CVC function and/or route for insertion. We report the history of a patient who developed SVC syndrome due to extensive extravascular tumor-related obstruction and who previously received implanted CVC for the purpose of chemotherapy. The endovascular approach consisted on treating venous obstructions while preserving the CVC function using bilateral asymmetric stenting and endovascular CVC-repositioning maneuvers.

## Case Presentation

### Patient

A 60-year-old male patient with central small-cell lung cancer having brain, adrenal gland, and bones metastasis (stage IV) underwent right-sided subclavian CVC implantation 3 months ago for a first-line chemotherapy (cisplatin and etoposide).

He was admitted to the emergency department with symptoms suggestive of SVC syndrome, including distended collateral neck veins, plethora of the face, and dyspnea.

Computed tomography (CT) chest venography evidenced central tumor growth with the obstruction of the SVC and the encirclement of both brachiocephalic veins (BCVs). Neither endoluminal component nor thrombus formation was identified. The left internal jugular vein (IJV) was larger than the right, corresponding to a major left venous return from the head and neck.

The diagnosis of subacute SVC syndrome related to tumor progression was established. To provide rapid relief of severe venous congestion and its associated morbidity, endovascular management was decided.

The CVC was still functional on radiographic evaluation. In an attempt to preserve the CVC for any subsequent chemotherapy, we opted to deploy two kissing stents in order to cover the SVC downstream and both BCVs. The CVC tip had to be moved into the ipsilateral BCV before stent placement. A written consent was obtained from the patient.

### Procedure

We performed the procedure in the radiology vascular-operating room using X-ray guidance under local anesthesia (10 mL of 1% lidocaine) with conscious sedation (intravenous administration of 1 mg midazolam). Standard physiological monitoring (pulse, blood pressure, oxygen saturation, and electrocardiogram) was carried out during the procedure. Antibiotic prophylaxis administrated parentally was 2 g cefazolin. Both the right and left femoral veins were cannulated with 80-cm long 10 French sheaths to facilitate the passage of large-diameter balloons and stents.

**Figure 1:**
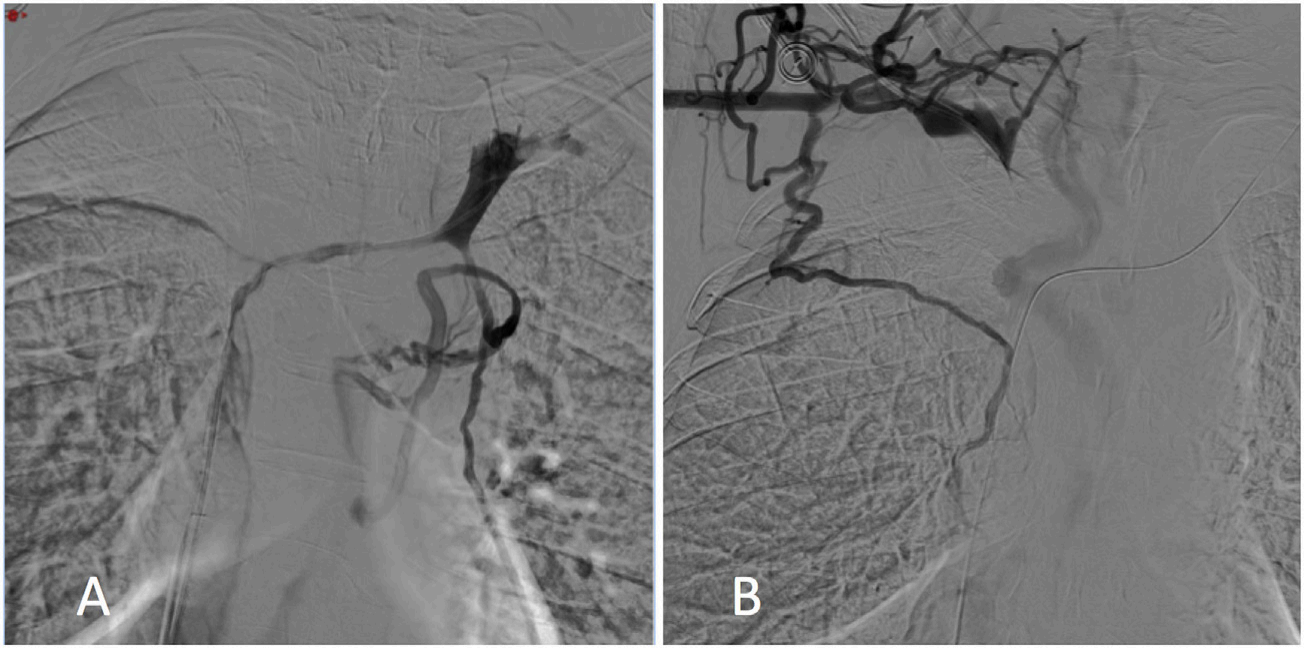
Digital subtraction phlebography from the left **(A)** and right **(B)** subclavian veins showed superior vena cava and both the right and left brachiocephalic vein stenosis with collateral channels.

The tight stenosis of the SVC and left BCV was crossed with a 5-Fr vertebral curve catheter (Terumo, Tokyo, Japan) and a 0.035-inch-angled hydrophilic guidewire (Terumo) *via* a femoral approach, and an SVC angiogram was carried out. Results showed a large filling defect of the SVC and left BCV stenosis with the presence of collateral veins (Figure 1A).

**Figure 2:**
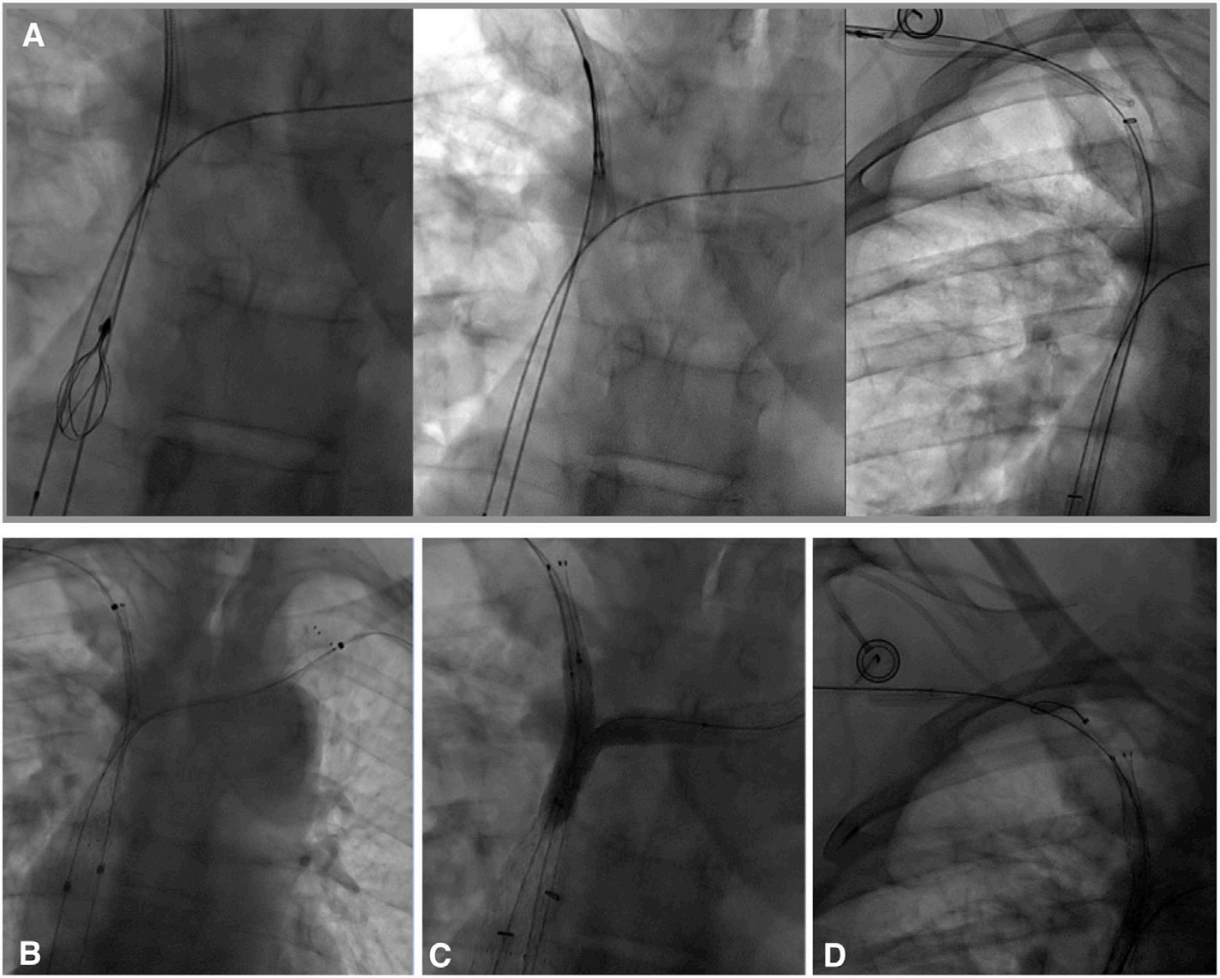
**(A)** In an attempt to preserve the central venous catheter (CVC) for further chemotherapy, the CVC tip was removed before stenting from the superior vena cava into the right subclavian vein using a trifoil EN-snare from access through the basilic vein. **(B)** Bilateral kissing stents deployment (a 14-mm nominal diameter and 150- and 100-mm nominal lengths, Sinus-XL Flex, Optimed) and **(C)** balloon angioplasty (an 8-mm diameter balloon within the right stent, a 10-mm diameter balloon within the left stent) of superior vena cava (SVC) and the bilateral caudal portion of the brachiocephalic veins were performed. **(D)** Using a snare-loop technique, the CVC tip was removed in the SVC within the stent to facilitate subsequent chemotherapy.

As it was not possible to cross the SVC and right BCV from the right femoral approach, the right basilic vein was punctured with a 20-G needle. A 45-cm long six-French introducer sheath was inserted and a phlebography was performed, revealing a subtotal stenosis of the right BCV in its central two-thirds with multiple collateral channels (Figure 1B). Mechanical recanalization of the right BCV and SVC occlusion was achieved with a 0.035-inch-angled hydrophilic guidewire from access through the basilic vein (Terumo) supported by a vertebral curve 5-French catheter (Terumo). When the guidewire had traversed the occluded segment, it was grasped with a one-loop snare on the opposite side from the femoral approach and retrieved through the hemostatic valve sheath in a “teleferic” mode. This allowed future insertion of a balloon or a stent over the guidewire. Once both BCVs have been recanalized, the tip of the CVC was snared and withdrawn into the right subclavian vein using a trifoil EN-snare (Merit Medical, South Jordan, USA) through a right basilic vein access (Figure 2A). Conventional percutaneous balloon angioplasty of the left BCV and SVC with a 4-mm diameter balloon was performed first (Passeo™ 35, Biotronik, Berlin, Germany). Bilateral kissing self-expandable Sinus-XL Flex stents (Optimed, Ettlingen, Germany) were deployed from the left BCV vein to the SVC (a 14-mm nominal diameter and 150 mm in length) and from the right BCV to the SVC (a 14-mm nominal diameter and 100 mm in length) (Figure 2B). The choice of stent diameters was guided by pretherapeutic data from CT with oversizing stents by up to 2-mm reference vessel diameter, in a non-involved BCV segment to help reduce delayed stent migration (8, 9). Kissing balloon angioplasty was then performed within the stents using a 10-mm diameter balloon (Armada™ 35, Abbott Vascular, Chicago, USA) into the left-sided stent and an 8-mm diameter balloon (Armada™ 35, Abbott Vascular) into the right-sided stent (Figure 2C).

Finally, the CVC was carefully replaced in the SVC within the stent (Figure 2D) using the same “snare-loop” technique.

**Figure 3:**
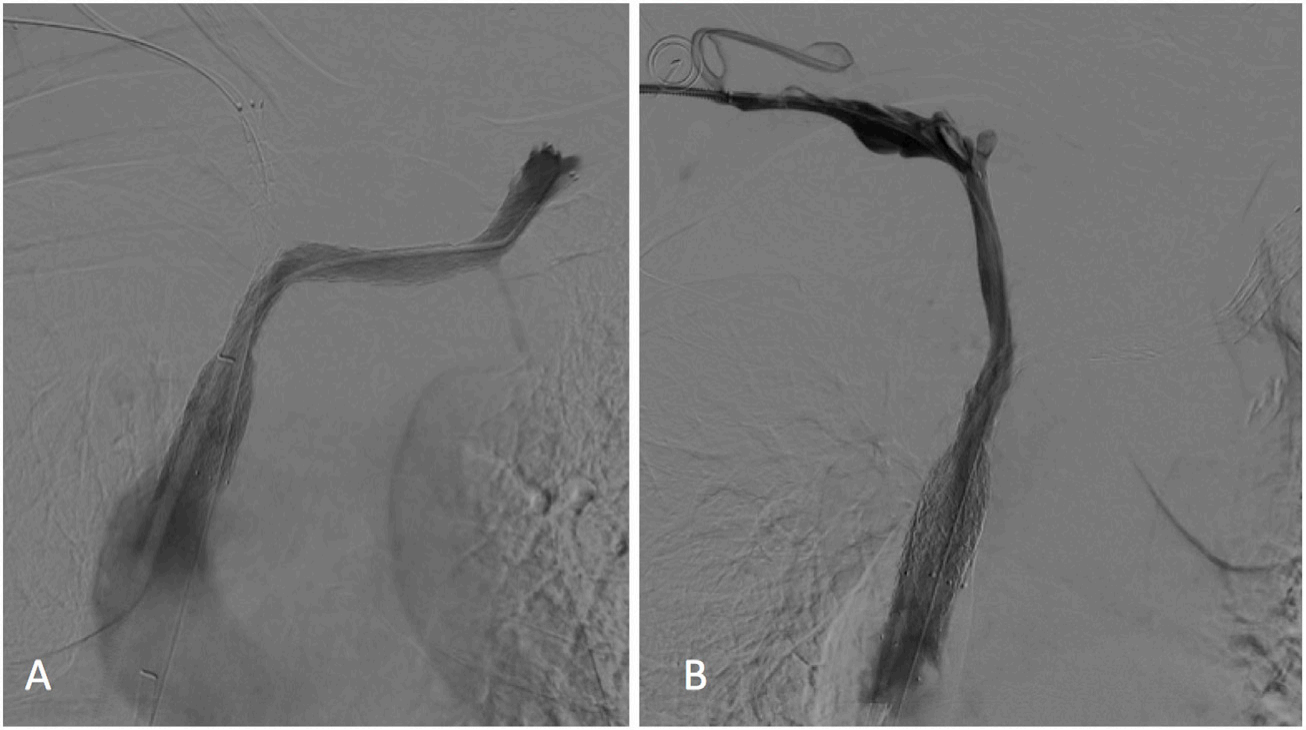
Left **(A)** and right **(B)** subclavian venograms obtained after stents placement showed restoration of superior vena cava and both brachiocephalic veins patency without collateral channels. No significant residual stenosis was observed within the stents.

A final cavogram showed improvement in the luminal diameter of the SVC and both BCVs with a free flow of contrast material into the right atrium and no collateral veins (Figure 3).

Intravenous 7,000-IU heparin (5,000-lU heparin bolus at the beginning of the procedure followed by an administration of 2,000 IU after balloon angioplasty) was given *via* femoral venous sheath during the procedure. The procedure was successfully completed within 2 h.

### Follow-up

The patient experienced quick relief of symptoms and was discharged home 48 h later, with a prescription of 60 mg enoxaparin and 100 mg/day acetylsalicylic acid for 3 months. No procedure-related complications occurred. The sixth round of chemotherapy (carboplatine and etoposide) was administrated a week after reca-nalization.

**Figure 4:**
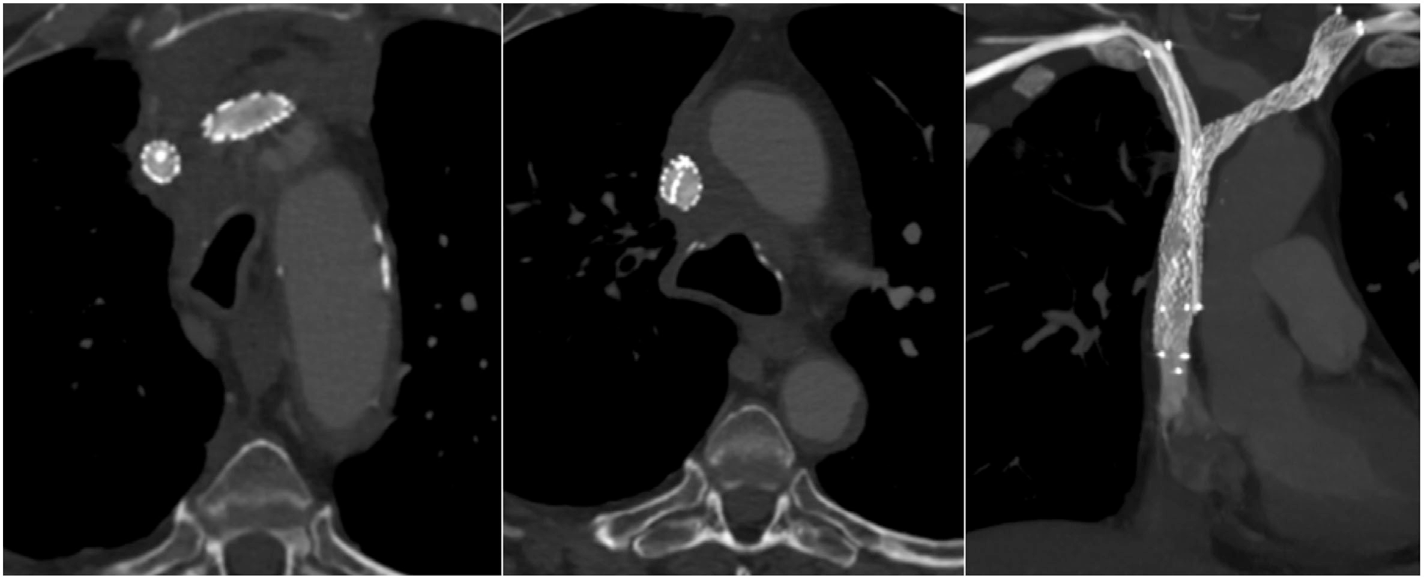
A 3-month follow-up computed tomography (axial sections and coronal section in maximum intensity projection) demonstrated bilateral stents patency without collateral veins. The left-sided stent, which ensures head and neck venous drainage, had a larger expansion than the right-sided one.

At a 3-month follow-up, the patient was asymptomatic without recurrence of his previous symptoms, and no mechanical problem of the CVC was observed. CT venography showed stents expansion and patency with no collateral drainage (Figure 4).

The patient died 4 months following the stenting procedure. The leading cause of death was neurologic complications related to brain metastasis.

## Discussion

The treatment of SVC syndrome using percutaneous transluminal stenting is a well-accepted technique with usually rapid symptom relief in these patients (10–12). The complications of this procedure including rupture of the SVC or stent migration are rare (12–14).

In our institution as in many centers, self-expanding bare-metal stents are the most common types of stents usually deployed in SVC obstructions (9, 12, 15, 16). Balloon-expandable stents are not recommended because of vein diameter differences between the distal- and proximal-landing zones and the short length of stents. Even if the use of covered stents versus bare-metal stents has suggested superior patency rates with covered stents after 12 months in malignant SVC obstruction (17), covered stents should be used with caution due to concerns of stent migration and covering venous collaterals, particularly if placing a covered stent across the brachiocephalic confluence. Slow-balloon pre-dilatation is required if the occlusive lesion precludes the passage of the stent delivery system. We also routinely perform post-stent dilatation if there is a >30% residual stenosis. This appears to be required in most of the patients (7, 15).

Data on the performance of bilateral stenting are limited. Dyet et al. stated that they had discontinued bilateral stent placement in SVC syndrome, having seen that unilateral placement was also clinically effective (16). Two studies found equally good initial clinical success in both groups, but a higher proportion of obstructive complications were seen in the bilateral group (18, 19). The bilateral technique is technically more demanding, and it is also more expensive and time-consuming.

When both BCVs are invaded by the tumor, unilateral BCV revascularization is sufficient and provides higher flow through the stent than when both BCVs are revascularized (20). The choice of recanalization side is based on the preferential head and neck venous drainage side or the inability to access both sides (1, 20, 21). CT phlebography is usually the modality of choice to determine the location of SVC obstruction and the involvement of BCV, and to provide an evaluation of head and neck venous drainage *via* internal jugular vein size (21). In our case, the left IJV vein was the major venous return from the head and neck, whereas the right IJV was thin. Therefore, the SVC downstream and the left BCV had to be recanalized to relieve symptoms.

Three options were considered. The first one was an SVC stenting with repositioning of the CVC just before and after stenting (1, 22, 23). CVCs are usually removed before stenting to prevent catheter fixation between the stent and the vessel wall with the associated risk of catheter dysfunction or catheter loss (24). To avoid catheter removal and the associated strain and risks of a new CVC insertion for the patient, a technique of repositioning before and after stenting was proposed, as previously described by Qanadli et al. (25). This option may have been considered in case of focal SVC stenosis without BCV veins involvement.

The second option would have been the SVC and left BCV stenting to assure head and neck venous drainage without preserving the CVC. This option may have led to catheter dysfunction or catheter loss. However, chemotherapy is also the standard form of treatment of malignant SVC syndrome. After rapid relief of symptoms after stenting, there is a need to continue the scheduled coadjuvant treatments (chemotherapy, radiotherapy, or both) (26). In this situation, unilateral stenting while preserving CVC access by leaving a peripherally inserted central catheter or an arm port CVC over the basilica vein access guidewire would have been another possibility.

A bilateral kissing stent deployment in order to cover the SVC downstream and both BCVs with CVC repositioning was the third and preferred option. This SVC reconstruction had two different objectives for each side. Left recanalization assured head and neck venous drainage, whereas right recanalization permitted CVC repositioning and subsequent in-stent replacement.

A last possibility is to deploy two different self-expandable stents, with a larger one in the SVC and left BCV. A larger stent would provide higher flow through it, reducing the risk of obstructive complications (27). In our case, the priority was to keep the left-sided flow patency to ensure head and neck drainage. We have oversized balloon angioplasty within the left-sided stent (a 10-mm diameter) comparatively to balloon angioplasty within the right-sided stent (an 8-mm diameter). Follow-up CT confirmed a larger expansion of the left-sided stent.

We reviewed published case reports of SVC stenting with catheter repositioning (Table 1). All previous cases reported CVC repositioning through the stent used in the treatment of SVC syndrome (22, 24, 25, 28, 29). Only two patients had SVC occlusion.

**Table 1:** Literature review of reported cases on technical differences of catheter repositioning during the treatment of SVC obstruction.

Patient sex/age (years)	CVC type	CVC position/tip	SVC obstruction stenosis/occlusion	BCV obstruction stenosis/occlusion	CVC-repositioning approach	Stenting SVC	Stenting RBCV/LBCV	SVC reconstruction
F/61 (22)	Hickmann	R SCV/NS	0/0	1/0	Ipsilateral IVJ	0	1/0	0
M/61 (22)	Hickmann	R SCV/NS	1/0	1/0	Ipsilateral IVJ	0	1/0	0
F/76 (22)	Hickmann	L IJV/NS	0/0	1/0	Ipsilateral CV	0	0/1	0
M/61 (22)	Hickmann	L IJV/NS	1/0	1/0	Ipsilateral CV	0	0/1	0
M/63 (22)	Hickmann	L IJV/NS	0/0	1/0	Ipsilateral CV	0	1/0	0
F/77 (22)	Hickmann	R IJV/NS	0/0	1/0	Ipsilateral CV	0	1/0	0
F/24 (22)	ICVC	L BCV/NS	1/0	0/1	Contralateral CV	1	0/0	0
M/51 (22)	ICVC	L BCV/NS	1/0	0/1	Contralateral CV	1	0/0	0
M/48 (24)	ICVC	L SCV/SVC	0/1	0/0	Ipsilateral BV	1	0/0	0
F/49 (25)	ICVC	R SCV/SVC	0/1	0/0	Ipsilateral BV	1	0/0	0
F/44 (28)	ICVC	L SCV/SVC	1/0	0/0	R femoral vein	1	0/0	0
M/54 (29)	ICVC	L SCV/SVC	1/0	0/1	R IJV + L IJV	1	0/1	0
M/60^a^	ICVC	R SCV/SVC	0/1	1/1	Ipsilateral BV + R femoral vein	2	1/1	1

*BCV, brachiocephalic vein; BV, basilica vein; CVC, central venous catheters; CV, cubital vein; ICVC, implanted central venous catheter; IJV, internal jugular vein; L, left; NS, not specified; R, right; SVC, superior vena cava*.

*^a^Refers to our article*.

Our case reports for the first time SVC and BCV reconstruction, for the purpose of not only treating venous obstruction but also preserving CVC function. Because of the bilateral involvement of BCV, one axis (one stent) was used to treat venous obstruction and the second one (one stent) was implanted to create a venous access and save CVC function.

## Concluding Remarks

In conclusion, even if unilateral stent placement is preferable in most patients with SVC syndrome because it is as clinically efficient as bilateral placement while offering lower cost, easier procedure, and lower rates of complications, bilateral stenting has to be considered in selected patients to avoid catheter loss or to facilitate subsequent catheter insertion. The procedure is safe and improves patients’ quality of life without the need of a second intervention for CVC placement.

## Ethics Statement

Written informed consent was obtained from the participant for the publication of this case report.

## Author Contributions

All authors listed have made a substantial, direct, and intellectual contribution to this work and approved it for publication.

## Conflict of Interest Statement

The authors declare that the research was conducted in the absence of any commercial or financial relationships that could be construed as a potential conflict of interest.
